# 2062. Impact of Indeterminate HIV Test Results on Efficacy of Emergency Department Routine HIV Screening

**DOI:** 10.1093/ofid/ofac492.1684

**Published:** 2022-12-15

**Authors:** Elizabeth A Aguilera, Gabriela P Del Bianco, Gilhen Rodriguez, Norma Perez, Gloria Heresi, James Murphy, Samuel J Prater

**Affiliations:** UTHealth, McGovern Medical School, Houston, Texas; UTHealth, McGovern Medical School, Houston, Texas; University of Texas McGovern Medical School, Houston, Texas; University of Texas McGovern Medical School, Houston, Texas; UTHealth, McGovern Medical School, Houston, Texas; UTHealth, McGovern Medical School, Houston, Texas; University of Texas Health Science Center at Houston, Houston, Texas

## Abstract

**Background:**

In 2019, 36,801 new HIV cases were reported in the United States. Emergency Department routine HIV testing is crucial to identify new asymptomatic HIV infections. Early diagnosis followed by prompt antiretroviral therapy decreases morbidity and mortality and reduces HIV transmission. The Emergency Department (ED) at Memorial Hermann Hospital (MHH) - Texas Medical Center (TMC) in Houston, Texas, implemented an ED-routine HIV screening program in June 2017. At times, the testing for HIV yields indeterminant results. Consequences of indeterminate HIV tests include individuals unaware of their HIV infection status transmitting infection and not receiving antiretroviral therapy.

**Methods:**

39,288 adults who presented to ED MHH –TMC from June 2017 to March 2022 with a Glasgow score > 9 were tested using an Opt-Out protocol for HIV infection. Testing comprised a screening assay (HIV 4^th^ Generation (GEN) ADVIA Centaur ^TM^ Ag/Ab COMBO (Siemens)) followed by a confirmatory test (Gennius^TM^ HIV1/HIV2). A second confirmatory test is performed if screening and first confirmatory tests yield conflicting (indeterminant) results (HIV1 RNA PCR or a repeat 4th GEN test).

**Results:**

824 (2.0 %) patients tested positive for HIV infection; 94 (0.2%) yielded indeterminate test results. 61 (64.8%) of the patients with indeterminate findings received confirmatory testing; 37 (39.4%) before leaving the hospital (35 HIV negative, 2 positive). Of the 57 (60.1%) who left the hospital before confirmatory testing, 24 (42.1%) were traced and tested (21 HIV negative, 3 positive). 33 (35.1%) were lost to follow-up (11 of whom were reported homeless or in unstable housing).

**Conclusion:**

The primary cause of failure to follow up testing on patients who tested indeterminant during an ED visit is a loss of contact with the patients after leaving the ED. In turn, failure to link these patients to HIV care relates to a failure to complete confirmatory testing before completion of the hospital visit. Therefore, the quantitative results presented will enable the assessment of the best deployment of resources in capturing those patients currently lost to follow-up.

**Disclosures:**

**Norma Perez, DO**, Theratecnologies, Inc: Advisor/Consultant.

